# Bioactive NIR‐II Light‐Responsive Shape Memory Composite Based on Cuprorivaite Nanosheets for Endometrial Regeneration

**DOI:** 10.1002/advs.202102220

**Published:** 2022-02-26

**Authors:** Chenle Dong, Chen Yang, Muhammad Rizwan Younis, Jing Zhang, Gang He, Xingdi Qiu, Lian‐Hua Fu, Dong‐Yang Zhang, Hao Wang, Wenli Hong, Jing Lin, Xueqing Wu, Peng Huang

**Affiliations:** ^1^ Department of Obstetrics and Gynecology The First Affiliated Hospital of Wenzhou Medical University Wenzhou Zhejiang 325000 China; ^2^ Department of Obstetrics and Gynecology Shenzhen University General Hospital Clinical Medical Academy Shenzhen University Shenzhen 518060 China; ^3^ Marshall Laboratory of Biomedical Engineering International Cancer Center Laboratory of Evolutionary Theranostics (LET) School of Biomedical Engineering Shenzhen University Health Science Center Shenzhen 518060 China; ^4^ Wenzhou Institute University of Chinese Academy of Sciences Wenzhou Zhejiang 325000 China; ^5^ Oujiang Laboratory Wenzhou Zhejiang 325000 China

**Keywords:** cuprorivaite nanosheets, endometrial regeneration, intrauterine adhesions, NIR‐II light‐responsive, shape memory

## Abstract

Intrauterine adhesions (IUAs) caused by mechanical damage or infection increase the risk of infertility in women. Although numerous physical barriers such as balloon or hydrogel are developed for the prevention of IUAs, the therapeutic efficacy is barely satisfactory due to limited endometrial healing, which may lead to recurrence. Herein, a second near‐infrared (NIR‐II) light‐responsive shape memory composite based on the combination of cuprorivaite (CaCuSi_4_O_10_) nanosheets (CUP NSs) as photothermal conversion agents and polymer poly(d,l‐lactide‐*co*‐trimethylene carbonate) (PT) as shape memory building blocks is developed. The as‐prepared CUP/PT composite possesses excellent shape memory performance under NIR‐II light, and the improved operational feasibility as an antiadhesion barrier for the treatment of IUAs. Moreover, the released ions (Cu, Si) can stimulate the endometrial regeneration due to the angiogenic bioactivity. This study provides a new strategy to prevent IUA and restore the injured endometrium relied on shape memory composite with enhanced tissues reconstruction ability.

## Graphical abstract

A second near‐infrared light‐responsive shape memory composite, which is composed of cuprorivaite (CaCuSi_4_O_10_) nanosheets as photothermal conversion agents and polymer poly(d,l‐lactide‐*co*‐trimethylene carbonate) as shape memory building blocks, is developed for intrauterine adhesions prevention and endometrial regeneration.

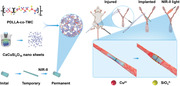

## 1 Introduction

The human endometrium is a dynamic tissue that undergoes periodic shedding and remodeling process.^[^ ^1^ ^]^ A healthy endometrial environment is indispensable for embryo implantation, while many intrauterine procedures such as spontaneous miscarriage, pregnancy curettage, endometrial polypectomy can lead to endometrial injury.^[^ ^2^ ^]^ The severe cases may result in intrauterine adhesions (IUAs), which is one of the main causes of female secondary infertility.^[^ ^3^ ^]^ Currently, there is no satisfactory therapy to cure IUAs as even the gold standard treatment, hysteroscopy adhesiolysis, holds great risk of recurrence (23% in moderate cases and 62% in severe cases) after surgery.^[^ ^4^ ^]^ Therefore, it is imperative to develop an effective strategy to prevent IUAs.

Physical antiadhesion barriers, including intrauterine devices, balloon, and hydrogels have been proved as effective treatments to separate the uterine walls.^[^ ^5^ ^]^ For example, Liu et al. have summarized the safety and efficacy of hyaluronic acid (HA) gels used as antiadhesion barriers after intraperitoneal or intrauterine surgery in clinical trials, which showed that HA gels could reduce the incidence of adhesions, especially for moderate cases.^[^ ^6^ ^]^ However, HA gels alone as physical barriers had a negligible effect on promoting reproductive performance or even failed to avoid the recurrence of IUAs due to the limited healing of injured endometrium.^[^ ^6^, ^7^ ^]^ To achieve the goal of IUA prevention and endometrial regeneration, comprehensive treatments based on antiadhesion barriers with the administration of drugs or stem cells have recently been proposed. For example, Yao et al. have developed a thermosensitive aloe‐poloxamer hydrogel embedded with steroid hormone (*β*‐estradiol) loaded nanoparticles for IUA treatment.^[^ ^8^ ^]^ Such composite hydrogel could not only preclude the readhesion, but also facilitate the endometrial regeneration due to the sustained release of *β*‐estradiol. However, the subsequent studies revealed that estrogen therapy is not suitable enough for patients with moderate to severe IUA.^[^ ^8^, ^9^ ^]^ Compared with estrogen therapy, cell therapy seems more attractive as the reported strategies using cells from a variety of sources (endometrial stromal cells, pluripotent stem cell‐derived mesenchymal stem cells, bone marrow mesenchymal stem cells, etc.) showed valuable endometrial remodeling ability.^[^ ^10^ ^]^ Whereas, the high cost and immunoreaction risk limit their application. Thus, the exploitation of antiadhesion implantations with enhanced endometrial regeneration ability is urgently demanded.

Shape memory polymers (SMPs) refer to a class of “smart” material, which can memorize a temporary shape in a predefined way and recover back to their original shape under an appropriate stimulation.^[^ ^11^ ^]^ Such a unique feature offers SMPs the possibility of biomedical deployments, including self‐expanding stents, biosensors, and self‐tightening sutures.^[^ ^12^ ^]^ For example, Radisic and co‐workers developed a minimally invasive approach to deliver functional engineered tissues using an elastic shape‐memory scaffold, which allow the tissue to collapse during injection, and subsequently regain its original shape at the desired location, suggesting that SMPs hold the potential advantages of minimal invasion, intelligent trigger, and ingredient delivery.^[^ ^12b^ ^]^ Such advantages would be beneficial for simultaneous IUA prevention and endometrial regeneration, although there is no report of SMPs for IUA prevention or endometrial regeneration. Moreover, by simply combining SMPs with photothermal conversion agents (PCAs), light‐triggered shape memory composites could be easily obtained since most of the SMPs could be triggered through temperature and PCAs could generate localized hyperthermia by transforming light energy into heat. However, most of the reported PCAs are restricted in the first near‐infrared (NIR‐I, 650–1000 nm) region. Compared to NIR‐I biological window, NIR‐II biological window (1000–1350 nm) is more attractive in the field of biomedical applications due to the deeper tissue penetration (>1 cm) and higher maximum permissible exposure.^[^ ^13^ ^]^ Recently, “Egyptian Blue Family” (XCuSi_4_O_10_, X represents Ca, Sr or Ba) has been exfoliated into nanosheets (NSs) due to their inherent layered structures, and the exfoliated NSs exhibited excellent photothermal‐conversion efficiency in both NIR‐I and NIR‐II region.^[^ ^14^ ^]^ More interestingly, these NSs are biodegradable and the bioactive Cu and Si ions could be sustainably released, which are powerful elements for stimulating new blood vessel formation.^[^ ^12^, ^14^, ^15^ ^]^ Since vascularization plays vital role in endometrial regeneration, XCuSi_4_O_10_ NSs‐based shape memory composite might be able to promote endometrial regeneration. Overall, XCuSi_4_O_10_ NSs‐based shape memory composite might be an alternative candidate for IUA treatment, which not only served as an NIR‐II light triggered antiadhesion barrier, but also simultaneously acted as a bioactive material for stimulating endometrial regeneration.

**Figure 1:**
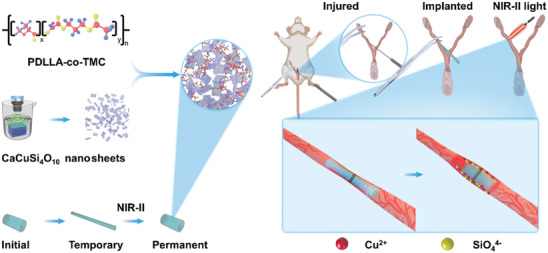
Schematic illustration of NIR‐II light‐responsive CUP/PT composites for IUA prevention and endometrial regeneration.

In this work, we developed an NIR‐II light‐responsive shape memory composite by integrating cuprorivaite (CaCuSi_4_O_10_) nanosheets (CUP NSs) with a widely used SMP: poly(d,l‐lactide‐*co*‐trimethylene carbonate) (PDLLA‐*co*‐TMC, denoted as PT)^[^ ^16^ ^]^ (**Figure** **1**). The shape memory performance of the as‐developed CUP/PT composites was evaluated along with their in vitro bioactivities using human endometrial epithelial cells (HEECs) and human umbilical vein endothelial cells (HUVECs). More importantly, we established an IUA‐induced rat model and investigated the therapeutic effects induced by the composites in vivo. This study provides a new avenue to prepare artificial intrauterine devices for preventing IUA and repairing injured endometrium, simultaneously.

## 2 Results and Discussion

### 2.1 Characterization of CUP NSs

**Figure 2:**
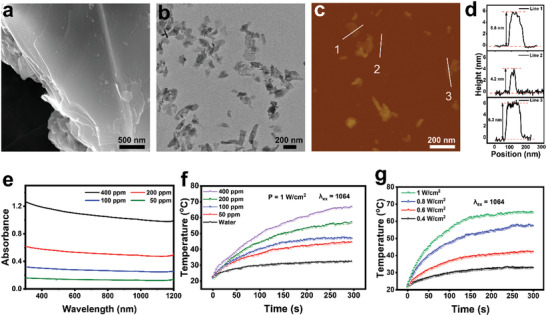
Characterization of 2D CUP NSs. a,b) SEM and TEM images of bulk CUP. c) AFM image and the corresponding height profile d) of exfoliated 2D CUP NSs. e) UV–vis/NIR absorption spectra of aqueous suspensions of CUP NSs with indicated concentrations. f) Concentration‐dependent photothermal property of CUP NSs aqueous dispersions under the mentioned irradiation conditions. g) NIR‐II laser power‐dependent photothermal property of CUP NSs (400 ppm).

CUP bulk powders were successfully synthesized following a conventional sol–gel method.^[^ ^15a^ ^]^ The phase purity was identified by X‐ray diffraction patterns (Figure S1, Supporting Information, JCPDS card No. 12‐0512), and the multilayer structure was confirmed by the scanning electron microscope (SEM) as shown in **Figure** **2**a. The CUP NSs were further delaminated by ultrasonic exfoliation of CUP bulk powder in deionized water (Figure 2b). The obtained CUP NSs were about 200 nm in size with a thickness of ≈4–6 nm (Figure 2c,d) as determined by the atomic force microscope (AFM) analysis. Such exfoliated CUP NSs exhibited broad absorption from ultraviolet (UV) to NIR‐II region (Figure 2e), indicating that CUP NSs might be utilized as NIR‐II PCA for the application of deep‐seated tissues such as uterus.

Subsequently, the concentration‐dependent photothermal property of CUP NSs was investigated under NIR‐II laser irradiation at a power density of 1.0 W cm^−2^ for 5 min (Figure 2f). A maximum temperature increment of 45.4 °C was observed in CUP NSs (400 ppm) under NIR‐II laser irradiation, while a much lower temperature elevation (24.7 °C) was recorded in pure water under the same irradiation conditions. Also, CUP NSs demonstrated laser power‐dependent photothermal property as an extensive hyperthermia was noticed under high power laser excitation (Figure 2g). Besides, the photothermal conversion efficiency of CUP NSs was calculated as 41.5%, implying the potential of CUP NSs as an efficient NIR‐II PCA.

### 2.2 Characterization of CUP/PT Composite

**Figure 3:**
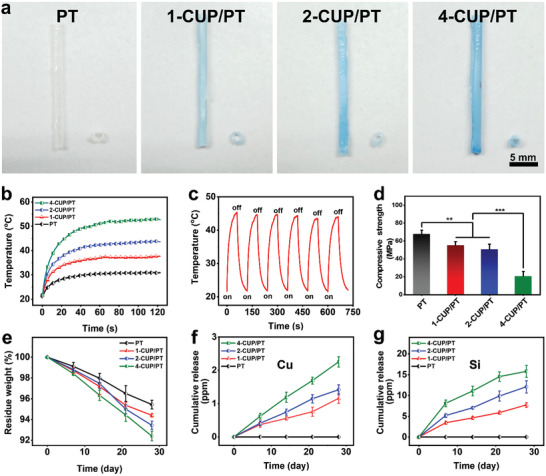
Characterization of CUP/PT composites. a) Photographs of tubular CUP/PT composites. b) NIR‐II photothermal properties of PT, 1‐CUP/PT, 2‐CUP/PT, and 4‐CUP/PT under 1064 nm laser (0.5 W cm^−2^) excitation for 2 min. c) Photothermal stability of the 2‐CUP/PT under six repetitive NIR‐II laser on/off cycles at a power density of 0.5 W cm^−2^. d) Compressive Young's moduli of different CUP/PT composites (*n* = 3). e) Degradation profile of the as‐mentioned CUP/PT composites in PBS buffer (*n* = 3). Cumulative release of f) Cu and g) Si ions from indicated CUP/PT composites (*n* = 3). All data are presented as mean ± SD. Statistical analysis was performed using one‐way ANOVA analysis, **p < 0.01, ***p < 0.001.

PDLLA‐*co*‐TMC (PT) is a typical SMP, which consists of both a soft part (PDLLA) and a hard part (TMC), respectively. The reasons for the selection of PT here is mainly ascribed to an excellent biocompatibility and the adjustable glass transition temperature (*T*_g_) value, which could be tailored to the temperature slightly higher than the body temperature via adjusting the ratio of PDLLA and TMC.^[^ ^12^, ^17^ ^]^ By physically combining PT with CUP NSs, a NIR‐II laser trigger shape memory composite (CUP/PT) was developed. As shown in **Figure** **3**a, tubular CUP/PT composites with different concentrations of CUP NSs (1, 2, and 4 wt%, designated as 1‐CUP/PT, 2‐CUP/PT, and 4‐CUP/PT, respectively) were prepared. The surface morphologies of all samples are shown in Figure S2 (Supporting Information). All surfaces were smooth and no apparent differences were observed between PT and CUP/PT composites since CUP NSs were embedded in the polymer network of PT. The Fourier transform infrared spectroscopy (FTIR) analysis (Figure S3, Supporting Information) confirmed the existence of CUP NSs in CUP/PT composite as stretching vibration of Si—O bond (791, and 1047 cm^−1^) and Cu—O bond (518, 591, and 659 cm^−1^) were observed. Also, the water contact angles of them were also at the same level (Figure S4, Supporting Information), implying that CUP NSs did not affect the surface energy of PT. Whereas, the incorporation of CUP NSs endowed CUP/PT composites with excellent NIR‐II photothermal performance, which was investigated under 1064 nm laser irradiation at a power density of 0.5 W cm^−2^ (Figure 3b). An obvious concentration‐dependent temperature increase was observed, while the maximum temperature elevation around 31 °C was recorded by 4‐CUP/PT composite. Moreover, no particular change was noticed after 6 repetitive NIR‐II laser on/off cycles, indicating the photothermal stability of CUP/PT composite (Figure 3c).

The effect of incorporated CUP NSs on the compressive mechanical property of CUP/PT composite was further studied. As shown in Figure 3d, compared to pure PT, the compressive strength of CUP/PT composite declined with the amount of doped CUP NSs, which may be ascribed to the interruption of inorganic particles in PT network. Despite the mechanical strength, the incorporated CUP NSs also affected the degradation rate of PT as all CUP/PT composites exhibited faster in vitro degradation as compared to PT, and the degradation rate was positively correlated with the amount of blended CUP NSs (Figure 3e). It is worth mentioning that the degradation of 2‐CUP/PT composite did not notably affect the photothermal performance (Figure S5, Supporting Information), which may be ascribed to the slow degradation process and the well‐distributed CUP NSs. More importantly, the bioactive Cu and Si ions could be sustainably released from CUP/PT composite, and the cumulative ion release concentrations were dependent on the amount of CUP (Figure 3f,g). As a control, no Cu and Si ions were found in pure PT. According to the previous studies, both Cu (0.064–6.4 ppm) and Si (11.9–47.4 ppm) ions within specific concentration could stimulate the angiogenic differentiation of HUVECs^[^ ^15^, ^18^ ^]^ and accelerate the wound healing process. Thus, CUP/PT composite might be a potential biodegradable candidate to regenerate injured endometrium.

### 2.3 Shape Memory Performance of CUP/PT Composite

**Figure 4:**
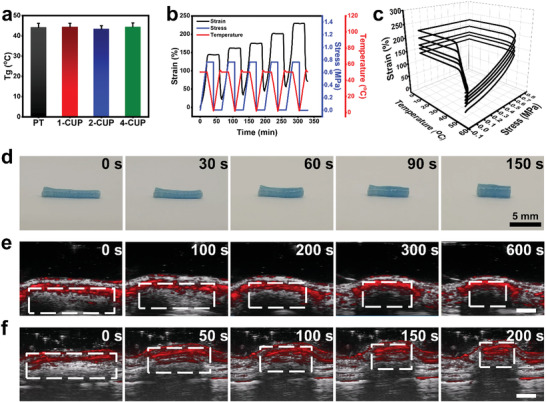
Shape memory performance of CUP/PT composites. a) *T*_g_ of PT and different CUP/PT composites from the DSC test (*n* = 3). b,c) 2D (b) and 3D (c) shape memory stress–strain–temperature curves of a representative 2‐CUP/PT composite from DMA testing. d) Shape recovery of a stretched tubular 2‐CUP/PT composite placed on a desk. e,f) Photoacoustic imaging of 2‐CUP/PT composite implanted underneath the skin (e), and into the isolated uterine lumen of a rat (f) under irradiation of NIR‐II light (0.5 W cm^−2^), respectively. Scale bar: 1.5 mm in (e) and 2.5 mm in (f). All data are presented as mean ± SD.

PT has been proved as a potential on‐demand biomedical candidate as its *T*_g_ is slightly higher than body temperature and the shape recovery process could be activated by an external thermal stimulus.^[^ ^16^, ^17^ ^]^ After the incorporation of different amounts of CUP NSs, negligible changes were seen in differential scanning calorimetry (DSC) curves (Figure S6, Supporting Information), and the quantitative analysis showed that all groups possessed approximately the same *T*_g_ value (≈44 °C, **Figure** **4**a), implying that CUP NSs had no significant effect on the activation temperature. Taking 2‐CUP/PT as an example, a cyclic shape memory test was performed up to five times using dynamic mechanical analysis (DMA) (Figure 4b,c). After the 5th cyclic shape memory test, the shape fixed rate (*R*_f_) and shape recovery rate (*R*_r_) of 2‐CUP/PT were as high as 99.55% and 92.16%, respectively (Table S1, Supporting Information,), indicating the good shape memory performance of CUP/PT composite under direct heat as an external stimulus.

To further investigate the shape memory behavior of CUP/PT composite under NIR‐II laser irradiation, a stretched tubular CUP/PT composite was fixed as the temporary shape at room temperature. After exposed to 1064 nm laser (0.5 W cm^−2^) for 150 s, the stretched tubular CUP/PT composite gradually turned back to the original shape (Figure 4d; Figure S7a, Supporting Information). To make a better presentation of the remote activation (NIR‐II laser), the stretched tubular CUP/PT composite was then implanted underneath the skin or into the isolated uterine lumen of a rat, respectively. The photoacoustic (PA) imaging system was applied to monitor the shape recovering process under NIR‐II laser irradiation (1064, 0.5 W cm^−2^). The wavelength of PA was identified based on the in vivo PA spectrum of CUP/PT composite (Figure S7b, Supporting Information). As shown in Figure 4e, stretched tubular CUP/PT composite inside subcutis returned to the original shape within 600 s, while the samples inside uterine lumen took 200 s (Figure 4f; Figure S7c, Supporting Information), which may be contributed to the easier operation of the isolated uterine lumen as compared to the living subcutis. Since the tissue penetration depth of NIR‐II light is usually larger than 1 cm, CUP/PT composite holds potential for the treatment of IUA in human using invasive optical fiber, or even as a noninvasive approach when a longer wavelength is applied. Such NIR‐II light‐responsive shape memory property endows CUP/PT composite with the potential as a “smart” antiadhesion barrier for IUA.

### 2.4 Bioactivity of CUP/PT Composite on HEECs and HUVECs In Vitro

**Figure 5:**
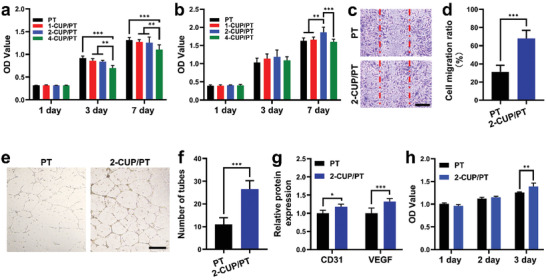
Bioactivities of different composites on HEECs and HUVECs. a,b) Proliferation of HEECs (a) and HUVECs (b) after culture on different CUP/PT composites for 1, 3, and 7 days (*n* = 4). c) Cell migration analysis using scratch method after cocultured with PT and 2‐CUP/PT composite for 16 h. d) The corresponding cell migration ratio (*n* = 3). e) The tube formation assessment of HUVECs after cocultured with PT and 2‐CUP/PT composite for 12 h. f) The corresponding number of formed tubes (*n* = 3). g) ELISA analysis of CD31 and VEGF from HUVECs after cocultured with PT and 2‐CUP/PT composite for 48 h (*n* = 5). h) Proliferation of HEECs after culture with medium collected from HUVECs study (*n* = 5). Scale bar: 500 µm. All data are presented as mean ± SD. Statistical analysis was performed using one‐way ANOVA analysis, **p < 0.01, ***p < 0.001.

HEECs played a prominent role in the preparation of blastocyst implantation, and have been widely used for the evaluation of endometrial regeneration. Whereas, HUVECs played a vital role in vascular homeostasis, and have been widely used for the evaluation of in vitro angiogenesis.^[^ ^19^ ^]^ The effects of CUP/PT composites on the proliferation of HEECs and HUVECs were conducted. As shown in **Figure** **5**a, HEECs were proliferated on both PT and CUP/PT composites, indicating the good biocompatibility. However, 4‐CUP/PT exhibited a significant impediment as compared to other groups at day 3 and day 7, which may be ascribed to the over‐released ions from 4‐CUP/PT as an excess concentration of Cu and Si ions would cause negative effects on cells.^[^ ^15a^ ^]^ A similar phenomenon also occurred in HUVECs as fewer cells were detected on 4‐CUP/PT composite after 7 days incubation (Figure 5b). It is worth mentioning that 2‐CUP/PT composite could significantly enhance the HUVEC proliferation as compared to other groups, and thus we chose 2‐CUP/PT composite as a representative composite sample for further cellular studies.

The effect of 2‐CUP/PT composites on HUVEC migration was evaluated through the in vitro scratch assay (Figure 5c,d). Compared with the PT group, more cells were migrated into the scratched wound in 2‐CUP/PT composite group after culture for 16 h as shown in Figure 5c. The quantitative analysis of cell migration ratio further confirmed the enhancement of 2‐CUP/PT composite on HUVEC migration (Figure 5d). The angiogenic capacity of 2‐CUP/PT composite was further evaluated using a tubular formation assay. The results are shown in Figure 5e,f, which indicated more tubes formation in Matrigel, when HUVECs were cocultured with 2‐CUP/PT composite than PT, suggesting that 2‐CUP/PT composite had a better effect on the pro‐angiogenesis of HUVECs. Moreover, the enzyme‐linked immunosorbent assay (ELISA) analysis (Figure 5g) showed that the 2‐CUP/PT composite significantly promoted more The expression of platelet endothelial cell adhesion molecule‐1 (PECAM‐1, CD31) and vascular endothelial growth factor (VEGF) secretion from HUVECs than PT, confirming the enhanced angiogenic bioactivity of 2‐CUP/PT composite. The mechanism behind this is mainly ascribed to the sustained release of Si and Cu ions as the plenty of previous studies have revealed that the released Cu or Si ions could promote the expression of hypoxia‐inducible factor‐1 (HIF‐1*α*) and VEGF, respectively, which further stimulate new blood vessel formation both in vitro and in vivo.^[^ ^18^, ^20^ ^]^ More interestingly, more HEECs proliferated after culture with medium collected from HUVECs/2‐CUP/PT system. This may be attributed to the higher VEGF secretion from HUVECs stimulated by 2‐CUP/PT since VEGF has been proved as an efficient growth and survival promoter for HEECs.^[^ ^21^ ^]^

To further verify the effect of released ions on the endometrial healing, we first evaluated the released ions with different concentrations from CUP NSs on cell viability similar to previous studies.^[^ ^15^, ^22^ ^]^ As shown in Figure S8a,b of the Supporting Information, both HUVECs and HEECs are suppressed in an extract medium of the high concentrations of CUP NSs, indicating that the over‐released ions cause cellular toxicity. Since the released ions from the CUP NSs (0.097–0.78 mg mL^−1^) were safe, the extract media were chosen for further investigation of cocultured HUVECs and HEECs. After culturing for 7 days, a significantly enhanced HEECs proliferation was observed in almost all the extract medium as compared to the control group (Figure S8c, Supporting Information), which was corresponding with the result of Figure 5h, implying the bioactive function of the released ions on endometrial healing. The upregulated VEGF in the coculture system further proved that an enhanced pro‐angiogenesis might make a key contribution to such promotion of the endometrial healing (Figure S8d, Supporting Information).

### 2.5 Endometrial Regeneration In Vivo by CUP/PT Composites

First, the in vivo biocompatibility of 2‐CUP/PT composite was evaluated through the biological toxicity examinations. The blood biochemical indexes (alanine aminotransferase (ALT), aspartate aminotransferase (AST), blood urea nitrogen (BUN), and creatinine (CREA)) verified the normal function of the liver and kidneys in all groups as no meaningful deviation was found (Figure S9, Supporting Information). Moreover, compared to the sham operation group, major organs (heart, liver, spleen, lungs, and kidneys) of rats in both PT and 2‐CUP/PT group did not show any noticeable abnormality (Figure S10, Supporting Information), suggesting the biosafety of implanted PT and 2‐CUP/PT composite.

**Figure 6:**
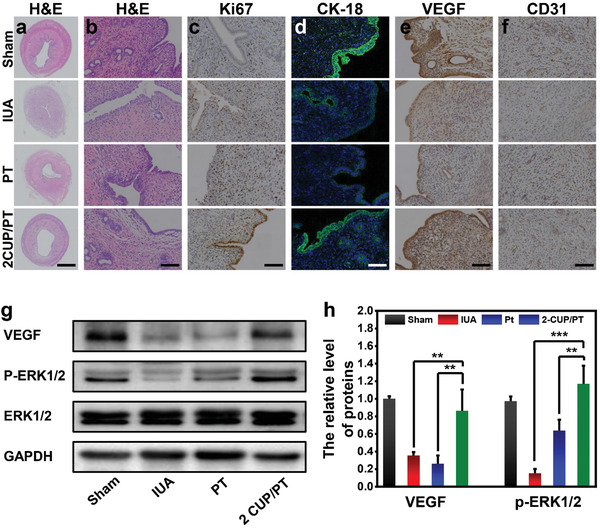
In vivo endometrium regeneration. a) Representative H&E staining images of uterine cavity in different treatments at day 14. b) The corresponding high magnification H&E images for the analysis of glands in each group. c,d) The immunohistochemistry staining targeting Ki67 (c) and immunofluorescence staining targeting CK‐18 (d) in different treatments at day 14. e,f) Representative images of immunohistochemistry staining targeting CD31 (e) and VEGF (f) in new‐formed tissues in different treatments at day 14. g) Western blotting analysis of the protein expressions of VEGF and P‐ERK1/2 in each group. h) The corresponding optical density analysis for western blotting assay (*n* = 5). Scale bar: 1 mm in (a), 50 µm in (b,c,e,f), and 10 µm in (d). All data are presented as mean ± SD. Statistical analysis was performed using one‐way ANOVA analysis, **p < 0.01, ***p < 0.001.

To further investigate the antiadhesion and endometrial regeneration ability of CUP/PT composite, a mechanically injured IUA rat model was established and treated with 2‐CUP/PT composite for 2 weeks. Hematoxylin & eosin (H&E) staining images (**Figure** **6**a,b) showed an obvious adhesion of uterine cavity in the group of IUA, while the large openings at the endometrial surface were displayed in both PT and 2‐CUP/PT composite, indicating their antiadhesion ability. However, more round or oval glands (Figure S11a, Supporting Information) were found in 2‐CUP/PT composite group as compared to PT group. The quantitative number of glands in 2‐CUP/PT composite group was about twofold higher than in PT group, suggesting the better repair efficiency of 2‐CUP/PT composite at the endometrial injury site. The antiadhesion and endometrial regeneration ability of 2‐CUP/PT composite were also investigated by comparing with clinical strategies (17*β*‐estradiol (E2) and HA gel). As shown in Figure S12 of the Supporting Information, the HA gel presented an excellent antiadhesion property but poor endometrial regeneration outcomes as compared to 2‐CUP/PT composites, while the Estrogen E2 displayed good endometrial regeneration property but poor antiadhesion than 2‐CUP/PT composite. The newly formed endometrial tissues were evaluated by assessing the expression of Ki67 in proliferative cells. As a nuclear antigen for cell proliferation at all stages, the expression of Ki67 would reflect the regeneration of the injured endometrium. The immunohistochemical staining (Figure 6c; Figure S11b, Supporting Information) displayed that Ki67 expressed in all groups, while 2‐CUP/PT had the highest Ki67 expression (>1‐fold than in PT) in the epithelial compartment, confirming the better repair efficiency of 2‐CUP/PT composite at the endometrial injury site. The immunofluorescence staining of CK‐18 (Figure 6d) further verified such conclusion as like sham operation group, 2‐CUP/PT group exhibited the same expression level of CK‐18 (a biomarker of epithelial tissue).

The immunohistochemistry staining of two angiogenic markers (VEGF and CD31) in new‐formed tissues was performed after 14 days (Figure 6e,f). More VEGF and CD31 were expressed in the group of 2‐CUP/PT composite as compared to IUA or PT group, which was verified by the quantitative analysis (Figure S11c,d, Supporting Information) as the expression of both VEGF and CD31 is upregulated by more than twofold. These results are in consistence with previous studies as Si‐contained or Cu‐contained biomaterials could stimulate in vivo angiogenesis due to the sustained release of bioactive Si and Cu ions, respectively.^[^ ^14^, ^15^, ^18^, ^23^ ^]^ More interestingly, it has been reported that VEGF could lead to downstream activation of the extracellular signal‐regulated kinase 1/2 (ERK1/2) signaling pathways, which play a vital role in several cellular processes, including survival, proliferation, migration, and differentiation.^[^ ^24^ ^]^ In the present study, the VEGF expression was decreased after the establishment of injured endometrium and recovered after treatment with 2‐CUP/PT (Figure 6g,h). Correspondingly, the expression of phosphorylated ERK1/2 (P‐ERK1/2) was significantly declined in IUA group, while an enhanced expression was noticed after an administration of 2‐CUP/PT, indicating an excellent endometrial regeneration activity of CUP/PT composite.

Moreover, the degradation of CUP/PT composite and the effect of the degradation process on the endometrial regeneration were investigated. As compared to the in vitro degradation (Figure 3e), both PT and 2‐CUP/PT composite exhibited faster degradation in vivo (Figure S13, Supporting Information), which may be ascribed to the complex pathological/physiological environment of the uterine cavity. Correspondingly, the repaired endometria of each group at different time points were evaluated (Figure S14, Supporting Information). Since negligible differences were noticed in the same group till 8‐weeks, which indicates that the degradation of the polymer has no distinct effects on the endometrial regeneration within 8 weeks. Taken together, we demonstrated that CUP/PT composite had a promising therapeutic potential to treat IUA. As far as the limitation of CUP/PT is concerned, in this work, we noticed a very slow degradation of CUP/PT, which requires an operation to take them out. In the future study, we would like to find more ideal degradable SMPs for the treatment of IUA, which could be completely degraded in about one month with at least 1–2 weeks’ shape retention depending on the healing rate of endometrium.

## 3 Conclusion

In summary, a bioactive NIR‐II‐responsive shape memory composite based on CUP NSs was successfully fabricated. The good photothermal and shape memory performance of CUP/PT composite in NIR‐II region endowed the as‐developed composite with promising potential as an intelligent antiadhesion barrier for IUA. Moreover, CUP/PT composite promoted both HEEC and HUVEC proliferation as well as the pro‐angiogenesis of HUVECs due to the sustained release of bioactive ions (Si and Cu). Further, in vivo study demonstrated the good antiadhesion ability of CUP/PT composite for IUAs and the remarkable wound healing property for injured endometrium. Our study provides a new avenue to develop shape memory composite based on therapeutic bioceramics for simultaneous IUA prevention and endometrial regeneration.

## 4 Experimental Section

### Materials

PDLLA‐*co*‐TMC copolymers (with a molar ratio of DLLA:TMC = 9:1) were purchased from ECO Biomaterials Co., Ltd. (Shenzhen, China). Calcium nitrate tetrahydrate (Ca(NO_3_)_2_·4H_2_O), copper nitrate hydrate (Cu(NO_3_)_2_·3H_2_O), and tetraethyl orthosilicate (TEOS) were provided by Aladdin Reagent Co., Ltd. (Shanghai, China). Antibodies against extracellular signal‐regulated kinase (ERK), phosphorylated‐ERK, and glyceraldehyde‐3‐phosphate dehydrogenase (GAPDH) were obtained from Cell Signaling Technology (CA, USA). VEGF antibody was ordered from Abcam (CB, UK). Cholecystokinin octapeptide (CCK‐8) assay kit was purchased from Beyotime Biotechnology (Shanghai, China). Other chemicals were bought from Servicebio Co., Ltd. (Wuhan, China) unless otherwise indicated.

### Synthesis of CUP Powders and CUP NSs

CUP powders were synthesized using a simple sol–gel method.^[^ ^15a^ ^]^ Briefly, TEOS, deionized water, and nitric acid were mixed in a molar ratio of 1:8:0.16, and then hydrolyzed under magnetic stirring for 30 min. Cu(NO_3_)_2_·3H_2_O and Ca(NO_3_)_2_·4H_2_O (molar ratio of Ca:Cu:Si = 1:1:4) were then added to form the final solution. The obtained transparent solution was kept at 120 °C for 48 h, then grounded, sieved, and calcined at 1000 °C. To prepare CUP NSs, 50 mg of the as‐obtained CUP powders were dispersed in 25 mL of milli‐Q water. The dispersion was ultrasonicated in an ice bath for 5 h using a power of 1000 W and then centrifuged for 10 min at 3000 rpm. The obtained supernatant was undergo centrifugation (4000–8000 rmp) for another 20 min and then collected for further use.

### Fabrication of CUP/PT Composites

The PLATMC and CUP NSs were dissolved in dichloromethane (DCM) under magnetic stirring at room temperature with different concentrations of CUP NSs (1, 2, and 4 wt%). The obtained mixture was transferred into a polytetrafluoroethylene mold to form a uniform film and placed in a hood for complete evaporation of DCM. The original CUP/PT tube was reshaped from the composite film by wrapping it on a glass stick.

### Characterization of CUP NSs and CUP/PT Composite

The morphology of CUP NSs was observed using transmission electron microscopy (TEM, HT7700, HITACH, Japan), while SEM (APREOS, FEI, the Netherlands) was used to characterize the morphology of CUP/PT composites. The thickness of CUP NSs was measured by an atomic force microscopy (AFM, Multimode‐8, Bruker, USA). The UV–vis spectra of CUP NSs dispersion were recorded using a spectrophotometer (Shimadzu, Japan). The *T*_g_ of CUP/PT composites was measured using a differential scanning calorimeter (DCUP, Netzsch Gerateban GmbH). FTIR spectra were recorded using a Perkin Elmer FTIR spectrometer (PerkinElmer, USA). The water contact angles of CUP/PT composites were measured using a contact angle goniometer (DSA100S, KRUSS, Germany). The degradation of CUP/PT composites was determined by calculating the weight loss in phosphate buffer saline (PBS) solution at different times (7, 14, 21, 28 days). The released Cu and Si ions from CUP/PT composites were measured using an Avio 200 ICP‐OES system (PerkinElmer Inc, USA). The compressive mechanical test of different cylinder samples (diameter of 8 mm) was conducted using an Instron machine (Instron‐5566, Instron, USA).

### Photothermal Performances of CUP NSs and CUP/PT Composite

Photothermal performance of the different concentrations of CUP NSs (50, 100, 200, and 400 ppm) and CUP/PT (0, 1, 2, and 4 wt%) was evaluated under NIR‐II laser (1064 nm) irradiation with different powers (0.4–1.0 W cm^−2^) for 5 min. The diameter of laser spot is ≈1 cm and the room temperature is ≈22 °C. The distance between the samples and the light source is about 1 cm. The real‐time temperature changes were recorded by an infrared thermal imaging camera (FLIR, CUP300, Arlington), and the photothermal stability was investigated through 6 repetitive NIR‐II laser on/off cycles. To investigate the relationship between the degradation and the photothermal performance, the degraded 2‐CUP/PT composite in PBS at different times were irradiated by an NIR‐II laser at a power density of 0.5 W cm^−2^ for 2 min. The real‐time temperature changes were recorded by an infrared thermal imaging camera. The photothermal conversion efficiency (*η*) was determined via the following equation

(1) $$\eta =\frac{hS\left(T_{m}-T_{s}\right)-Q}{I\left(1-10^{-A}\right)}$$

where *h* refers the heat transfer coefficient, *S* refers the surface area of the container, *T*_m_ refers to the equilibrium temperature, *T*_s_ refers to the surrounding temperature, *Q* refers the heat generated by the container and water under laser irradiation, *I* refers the laser power, and A refers the absorbance of CUP NSs at 1064 nm, respectively.

### Shape Memory Performance of CUP/PT Composite

The shape memory property of the CUP/PT films was examined by a DMA (TA Instruments Q800, USA). Briefly, the film was cut into 10 × 5 × 0.16 mm dimension and heated at 46 °C (slightly higher than *T*_g_) for 3 min to obtain an initial strain (*ε*_initial_). While, the stress was raised to 0.5 MPa at a stress rate of 0.025 MPa min^−1^ under a constant temperature (46 °C) to obtain a deformed strain (*ε*_deformed_). Then, the temperature was cooled to 0 °C at a rate of 2.3 °C min^−1^ and the stress was released back to 0 MPa to obtain a fixed stain (*ε*_fixed_). Finally, the temperature was increased to 46 °C at a rate of 2.3 °C min^−1^ to obtain a recovery strain (*ε*_final_) and equilibrated at 46 °C for 3 min. The same procedure was repeated for five times. The shape fixed rate (*R*_f_) and shape recovery rate (*R*_r_) were defined as follows

(2) $$R_{f}=\frac{\varepsilon_{\mathrm{fixed}}}{\varepsilon_{\mathrm{deformed}}}\times 100\%$$

(3) $$R_{r}=\frac{\varepsilon_{\mathrm{deformed}}-\varepsilon_{\mathrm{final}}}{\varepsilon_{\mathrm{deformed}}-\varepsilon_{\mathrm{initial}}}\times 100\%$$

Also, the shape memory behavior of CUP/PT tube was investigated using a stretching mode. Briefly, the original tube was stretched into a temporary shape at 80 °C and quickly moved to room temperature to obtain the temporary shape. Then, the fixed temporary stretched tube was placed on a desk, implanted beneath the skin or into the isolated uterine lumen of a rat, respectively. The recovery performances were recorded by a digital camera or PA imaging system (LAZR‐X, FUJIFILM VisualSonics, Inc., Canada) under the irradiation of NIR‐II laser (0.5 W cm^−2^). The distance between the samples and the light source is about 1 cm. The parameters for PA imaging are: depth (15.0 mm), width (14.0 mm), wavelength (1200 nm), frequency (40 MHz), PA gain (49.0 dB), and B‐mode gain (32.0 dB).

### In Vitro Cell Study

The primary HEECs were extracted from human endometrial tissues after induced abortion (Ethics Clearance No.: AEWC‐2021019). In brief, the endometrium was washed with PBS (three times) and cut into small pieces. Then, the tissues were digested with 2% collagenase in a horizontal shaking incubator at 37 °C for 70–80 min, while the tissues digestion was stopped by adding DMEM/F‐12 culture medium. After removing the impurities using a 100 *μ*m cell sieve, the endometrial cells were retained by a 40 *μ*m cell sieve. The obtained primary HEECs were cultured in epithelial cell media (EpiCM, ScienCell Research Laboratories, CA, USA) with the supplement of 5% CO_2_ in a humidified incubator (Thermo, San Diego, CA, USA) at 37 °C. The HUVECs were purchased from the ScienCell Research Laboratories (CA, USA) and cultured with corresponding endothelial cell medium (ECM).

For cell proliferation study, 0.5 × 10^4^ cells were seeded on CUP/PT composites in 48‐well plates and cultured for 1, 3, and 7 days. The cell viability of each time point was determined using a CCK‐8 assay kit. The cell migration experiment was conducted using a typical scratch assay. Briefly, 2 × 10^4^ HUVECs were seeded in a 24‐well plate and a tip‐induced wound was obtained across the diameter of each plate. After cocultured with CUP/PT composite for 16 h, the migrated cells were stained with crystal violet (0.1% w/v) and observed by a microscope (ECLIPSE Ti2, Nikon, Tokyo, Japan). The migration ratio was determined as wound healing area versus premigratory scratched open area using the ImageJ software (National Institutes of Health, USA). The in vitro vessel formation test was conducted using a Matrigel‐coated plate. HUVECs (2 × 10^4^) were cocultured with CUP/PT composites for 12 h, and the formed tubes were observed and calculated by a microscope and the ImageJ software (National Institutes of Health, USA), respectively. For ELISA, the medium from HUVECs cultured with PT and CUP/PT were collected after 48 h. The expression of platelet endothelial cell adhesion molecule‐1 (PECAM‐1, CD31) and VEGF were quantified using ELISA kits (Mlbio, Shanghai, China). To further verify the effect of the released ions on the endometrial healing, different concentration of CUP NSs (0.097–100 mg mL^−1^) were soaked in serum‐free ECM and incubated at 37 °C for 24 h. The suspension was then centrifuged, and the collected supernatant was sterilized using a filter (Millipore, 0.22 µm). The obtained medium was added with 10% fetal bovine serum and 1% P/S (penicillin/streptomycin), respectively. A transwell method was applied for the cell coculture study as HUVECs were cultured in the upper room and HEECs were cultured in the bottom room.

### Animal Models of IUA

A rat IUA model was established based on a mechanically injured model.^[^ ^25^ ^]^ Briefly, healthy Sprague‐Dawley female rats (7–8 weeks old) were purchased from Guangdong Laboratory Animal Co., Ltd. (Guangdong, China) and anesthetized with 10% chloral hydrate (250 mg kg^−1^). The abdominal cavity was opened, and a 2 mm incision was made in the left uterus. The endometrial damage was generated using a scraping spoon to repeatedly scratch the uterus until rough and grainy uterine wall was felt. After surgery, the rats were randomly divided into four groups and defined as IUA group, sham operation group, PT group, and 2‐CUP/PT group. Both IUA and sham operation group did not receive any treatment except the uterine was scratched in IUA group. For the groups of PT and 2‐CUP/PT, temporary PT or 2‐CUP/PT, tubes were implanted into the injured uterine lumens. The temporary tubes were recovered to the initial tubes by an external heating lamp or 1064 nm laser irradiation at a density of 0.5 W cm^−2^ for 2 min. The distance between the samples and the light source is about 1 cm. All rats were sacrificed at 14 days postsurgery, and uterine tissues were collected and fixed for further histological examination. Another similar animal experiment was carried out by using clinical products as the control groups: E2 and HA gel. After the rat IUA model was built, 50 µL of 17*β*‐estradiol solution (10 µg mL^−1^) and HA gel were injected into an injured uterine cavity to set up HA and E2 group, respectively. The studies were approved by the Animal Ethical and Welfare Committee of Shenzhen University (AEWC‐SZU). All experimental procedures were performed according to the Guide for the Animal Care and Use of Laboratory.

### Biological Toxicity Examination

The major organs of the rats were collected for H&E histological analysis, while 0.8 mL blood was harvested from the rats for blood biochemical analysis of ALT, AST, BUN, and CREA.

### Histological, Immunohistochemical, and Immunofluorescence Analysis

Samples were fixed in 10% formaldehyde for 24 h, and then embedded in paraffin and cut into different sections. Subsequently, the sections of each sample were stained with H&E. A slide scan system (SQS1000, TEKSQRAY, Shenzhen, China) was used to observe the histomorphology. For immunohistochemical analysis, the samples were dewaxed and rehydrated using 0.3 vol % hydrogen peroxide. The obtained samples were blocked using 5% bovine serum albumin (BSA) for 30 min at 37 °C. Subsequently, the sections were incubated with primary antibodies, including Ki67, CD31, and VEGF for overnight at 4 °C. After treating with the horseradish peroxidase (HRP)‐conjugated secondary antibody, the sections were stained with hematoxylin, captured using the SQS1000slide scan system, and analyzed by ImageJ. For immunofluorescence staining, the sections were rehydrated, blocked with 1.5% goat serum, and incubated with primary antibody of plasma cytokeratin 18 (CK‐18) for overnight at 4 °C. The obtained slice was incubated with HRP‐conjugated secondary antibody and diamidine phenylindole dihydrochloride. The images were acquired using a confocal microscopy (ZEISS, Jena, Germany).

### Western Blot Analysis

For western blot assay, a certain amount of uterine tissue was taken and grounded into powder in liquid nitrogen. The obtained tissue powder was lysed in Ripa buffer. Samples with equal amounts of total proteins were separated by sodium dodecyl sulfate‐polyacrylamide gel electrophoresis and then transferred to poly(vinylidene fluoride) membrane (Bio rad Laboratories, Hercules, CA, USA). The membrane was sealed with 5% BSA and then incubated with primary antibodies: VEGF, ERK, phosphorylated‐ERK, and GAPDH for overnight at 4 °C. Finally, the membranes were incubated with HRP‐conjugated secondary antibody and visualized using a chemiluminescence blotting detection system (Fluor Chem E, Protein Simple, Santa Clara, USA).

### In Vivo Degradation

To determine the in vivo degradation of the materials as well as the effect of degradation on the endometrial regeneration, both PT and 2‐CUP/PT composite were positioned in the injured uterus of the IUA rats for 2, 4, 6, and 8 weeks. The samples were taken out and weighed at each time point. Also, the repaired endometria were collected, fixed in 10% formaldehyde, and embedded in paraffin for H&E staining.

### Statistical Analysis

All data were expressed as the means ± standard deviation (SD), which was performed following the one‐way ANOVA analysis model using GraphPad Prism 9 software. A *p*‐value < 0.05 was considered statistically significant.

## Conflict of Interest

The authors declare no conflict of interest.

## Supporting information

Supporting Information

## Data Availability

Research data are not shared.
