# Supplementary material for: Bioactive NIR‐II Light‐Responsive Shape Memory Composite Based on Cuprorivaite Nanosheets for Endometrial Regeneration
Source: Adv Sci (Weinh). 2022 Feb 26;9(12):2102220. doi: 10.1002/advs.202102220 (PMC9036008; doi:10.1002/advs.202102220)
Supplement: Supplementary file 1 — Supporting Information [file ADVS-9-2102220-s001.pdf]

ADVANCED SCIENCE
Open Access

Supporting Information

for *Adv. Sci.*, DOI 10.1002/advs.202102220

Bioactive NIR-II Light-Responsive Shape Memory Composite Based on Cuprorivaite Nanosheets for Endometrial Regeneration

Chenle Dong, Chen Yang, Muhammad Rizwan Younis, Jing Zhang, Gang He, Xingdi Qiu, Lian-Hua Fu, Dong-Yang Zhang, Hao Wang, Wenli Hong, Jing Lin, Xueqing Wu and Peng Huang**

DOI: 10.1002/advs.202102220
Article type: Full Paper

Bioactive NIR-II light-responsive shape memory composite based on cuprorivaite nanosheets for endometrial regeneration

Chenle Dong, Chen Yang, Muhammad Rizwan Younis, Jing Zhang, Gang He, Xingdi Qiu , Lian-Hua Fu, Dong-Yang Zhang, Hao Wang, Wenli Hong, Jing Lin, Xueqing Wu, Peng Huang**

C. Dong, X. Qiu, H. Wang, W. Hong, Prof. X. Wu, and Prof. P. Huang
Department of Obstetrics and Gynecology, The First Affiliated Hospital of Wenzhou Medical University, Wenzhou, Zhejiang, 325000, China;
E-mail: sughfck@szu.edu.cn

C. Dong, X. Qiu, H. Wang, W. Hong, and Prof. X. Wu
Department of Obstetrics and Gynecology, Shenzhen University General Hospital, Clinical Medical Academy, Shenzhen University, Shenzhen 518060, China.

C. Dong, Dr. C. Yang, Dr. M. R.Younis, G. He, Dr. L. Fu, Dr. D. Zhang, Prof. J. Lin, and Prof. P. Huang
Marshall Laboratory of Biomedical Engineering, International Cancer Center, Laboratory of Evolutionary Theranostics (LET), School of Biomedical Engineering, Shenzhen University Health Science Center, Shenzhen 518060, China.
Email: peng.huang@szu.edu.cn

Dr. C. Yang
Wenzhou Institute, University of Chinese Academy of Sciences, Wenzhou, Zhejiang 325000, China.
Oujiang Laboratory, Wenzhou, Zhejiang 325000, China.

Table 1. Shape memory parameters of 2-CUP/PT with 5 cyclic tests using dynamic mechanical analysis (DMA).

Cycle	ε_{intia} (%)	$\varepsilon_{deformed}$ (%)	ε_{fixed} (%)	ε_{final} (%)	R_f (%)	R_r (%)
1	0.36	143.75	143.28	21.75	99.67	85.08
2	21.75	161.45	161.01	33.57	99.73	91.41
3	33.57	174.13	173.70	43.99	99.75	92.59
4	43.99	200.88	200.45	56.24	99.79	92.19
5	56.24	229.04	228.01	69.78	99.55	92.16

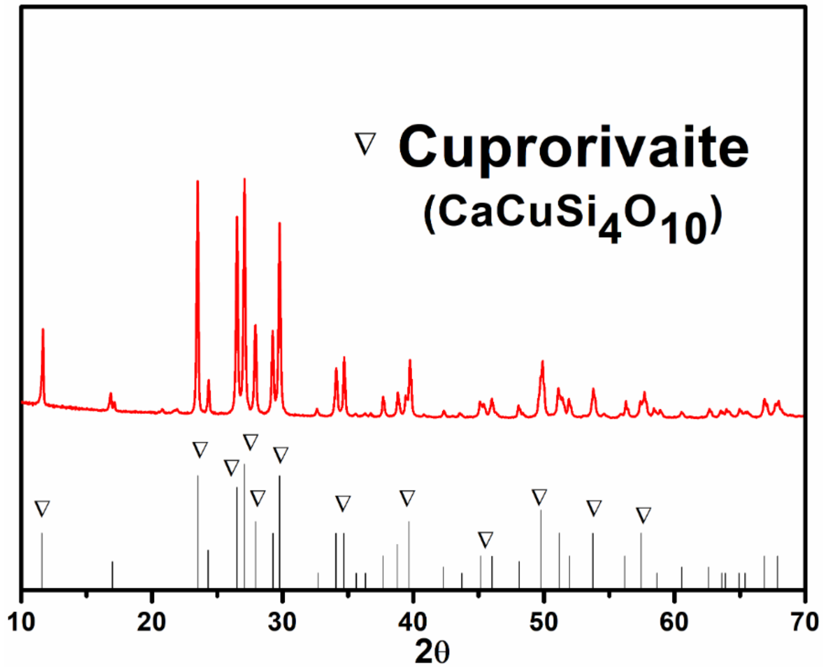

Figure S1. XRD spectrum of cuprorivaite ($CaCuSi_4O_{10}$) nanosheets (CUP NSs).

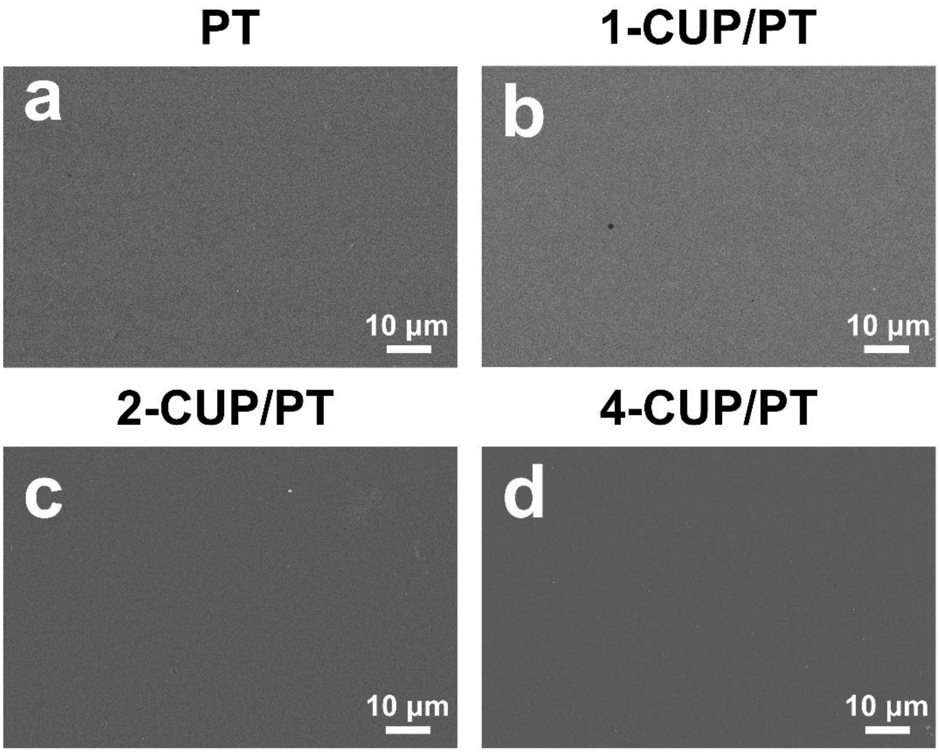

Figure S2. Scanning electron microscope (SEM) images of different composites: (a) PT, (b) 1-CUP/PT, (c) 2-CUP/PT, and (d) 4-CUP/PT. Scale bar: 10 μm.

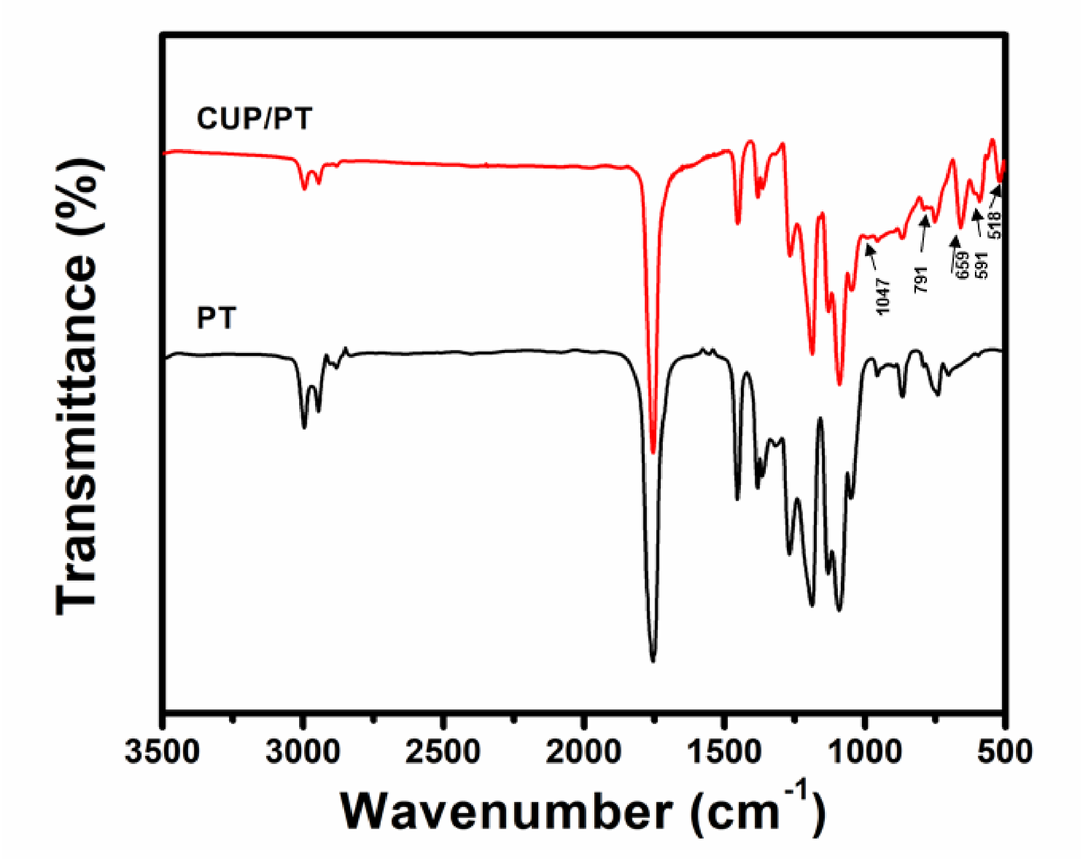

Figure S3. Fourier transform infrared spectroscopy (FTIR) spectra of PT and CUP/PT composites.

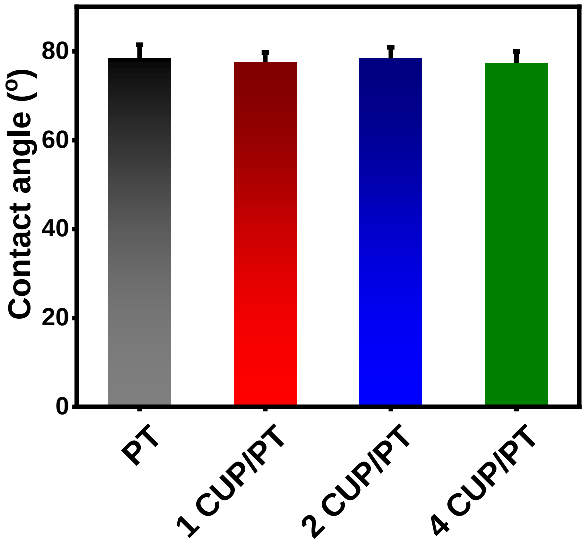

Figure S4. Water contact angles of different composites (PT, 1-CUP/PT, 2-CUP/PT, and 4-CUP/PT). All data are presented as mean ± SD, n=3.

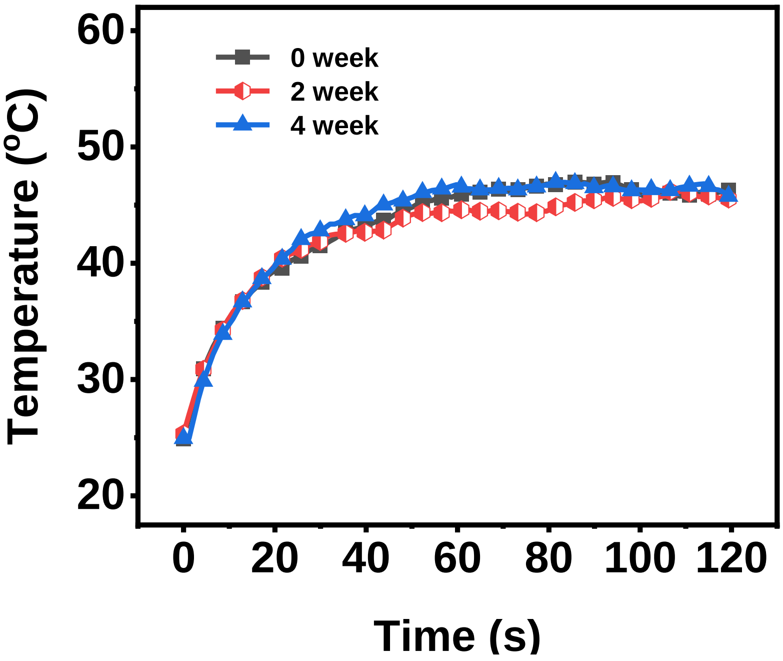

Figure S5. Photothermal performances of 2-CUP/PT composites after *in vitro* degradation over time.

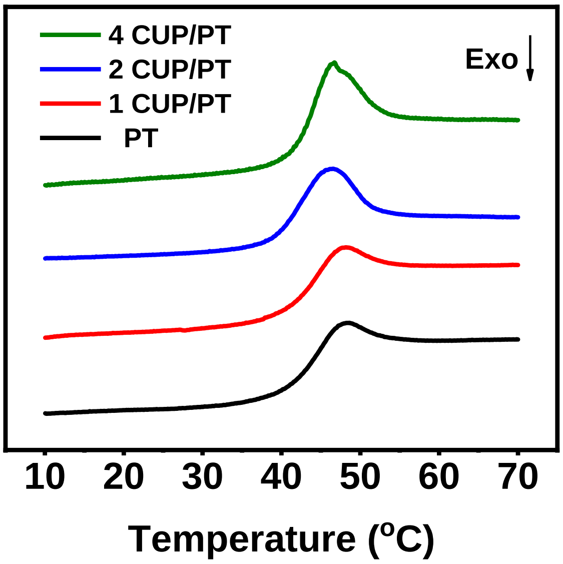

Figure S6. DSC curves of different composites (PT, 1-CUP/PT, 2-CUP/PT, and 4-CUP/PT).

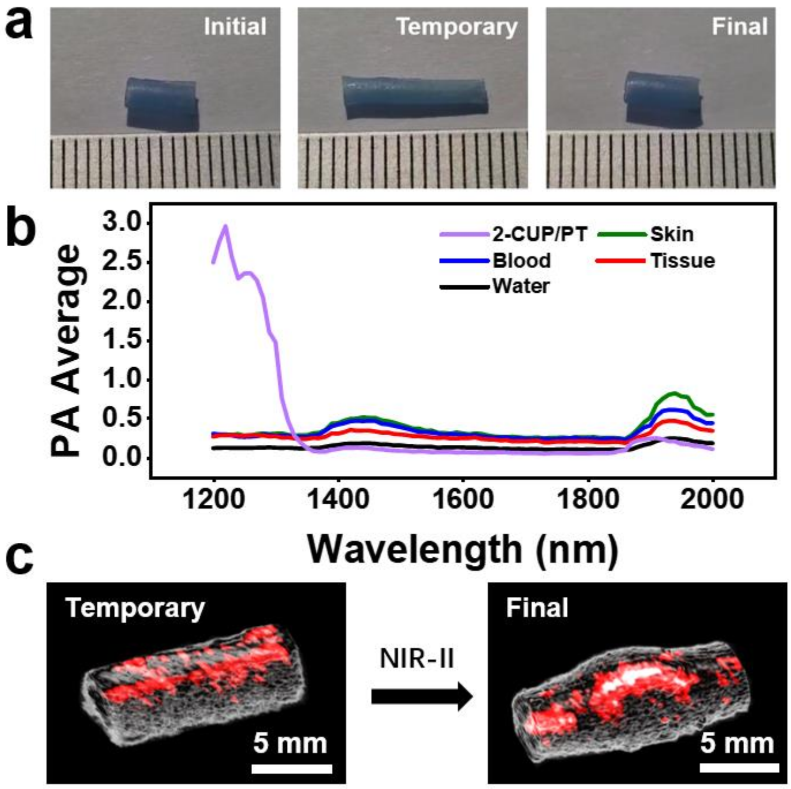

Figure S7. a) Representative photographs of 2-CUP/PT tube before and after NIR-II laser irradiation. b) NIR-II PA spectra of 2-CUP/PT composite from 1200 to 2000 nm. c) 3D reconstructed PA images of 2-CUP/PT tube implanted in isolated uterine lumen of rat before and after NIR-II laser irradiation. Scale bar: 5 mm.

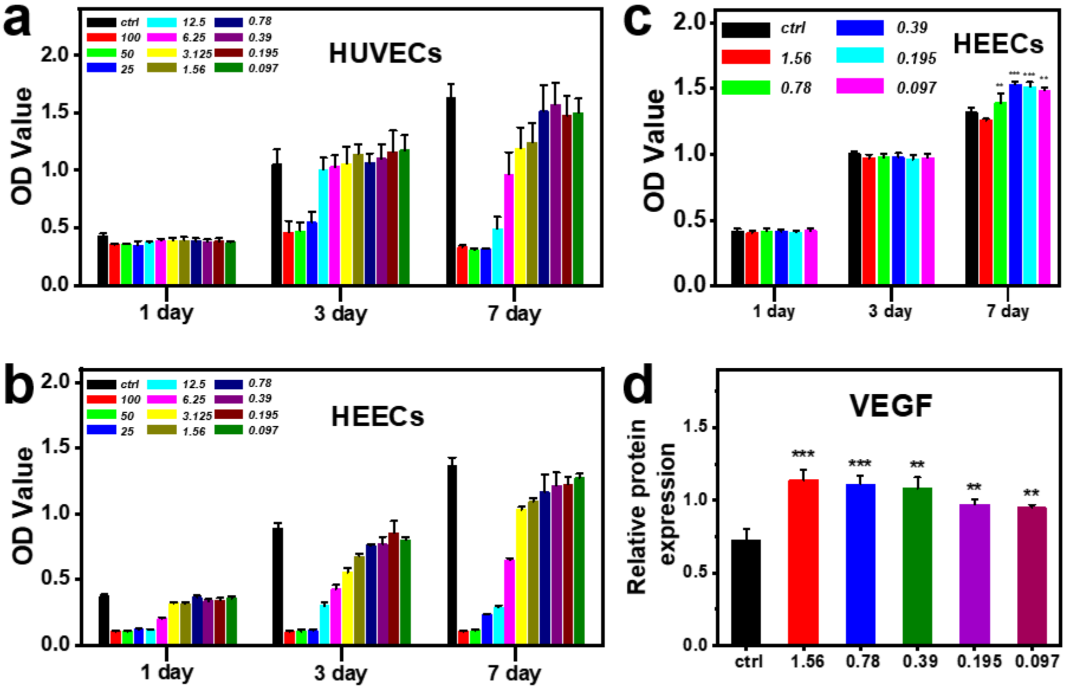

Figure S8. Bioactivity of released ions from CUP on HEECs and HUVECs. a, b) Proliferation of HUVECs (a) and HEECs (b) after culture with different concentrations of released ions from CUP NSs for 1, 3, and 7 days (n=5). c, d) Proliferation of HEECs (n=3) (c) and the relative VEGF expressions (n=5). (d) after co-culturing HUVECs and HEECs with different concentrations of released ions from CUP NSs. (*p < 0.05, **p < 0.01, ***p < 0.001 vs. ctrl (control group)). All data are presented as mean ± SD. Statistical analysis was performed using one-way ANOVA analysis, ***p*< 0.01 vs. ctrl, ****p*< 0.001 vs. ctrl.

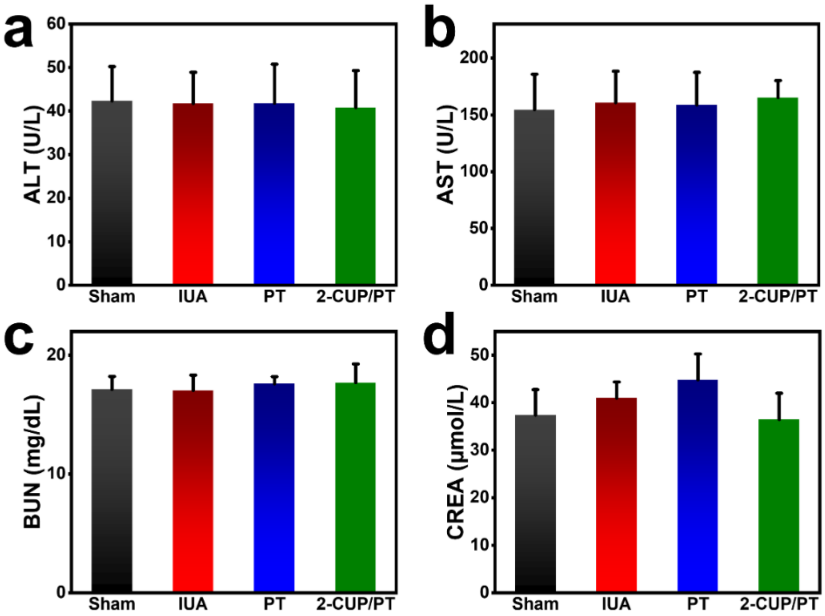

Figure S9. Blood biochemistry analysis of ALT (a), AST (b), BUN (c), and CRE (d) of the rats with different treatments at day 14. All data are presented as mean ± SD, n=3.

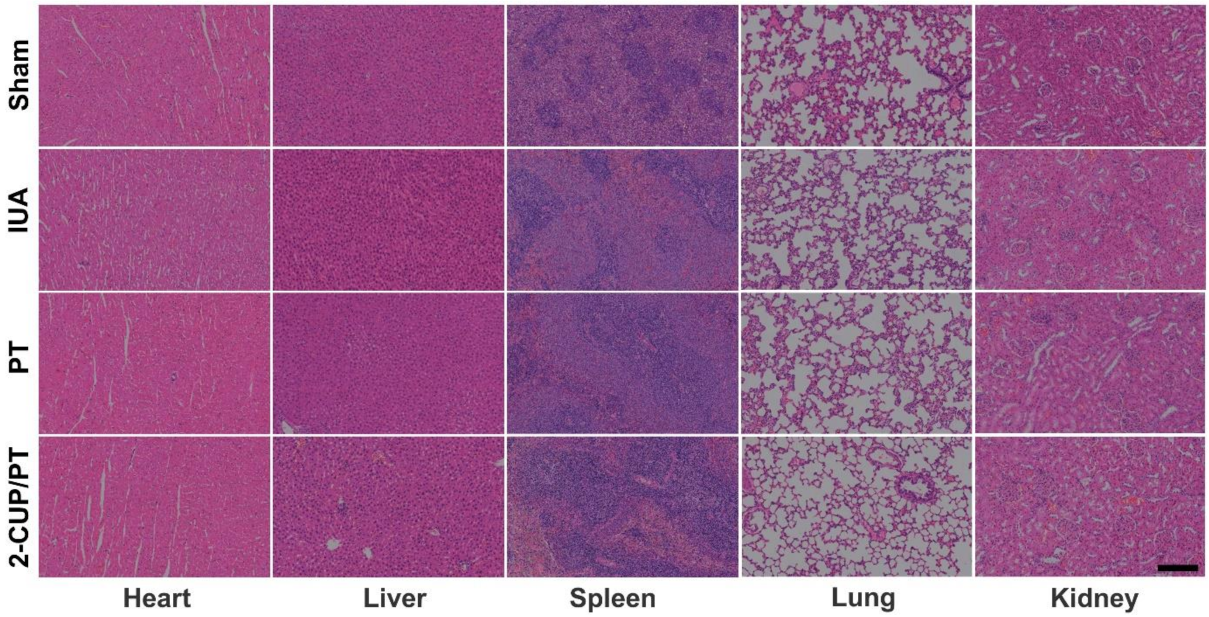

Figure S10. Representative H&E staining images of major organs (heart, liver, spleen, lung, and kidneys) from mice with different treatments at day 14. Scale bar: 200 μm.

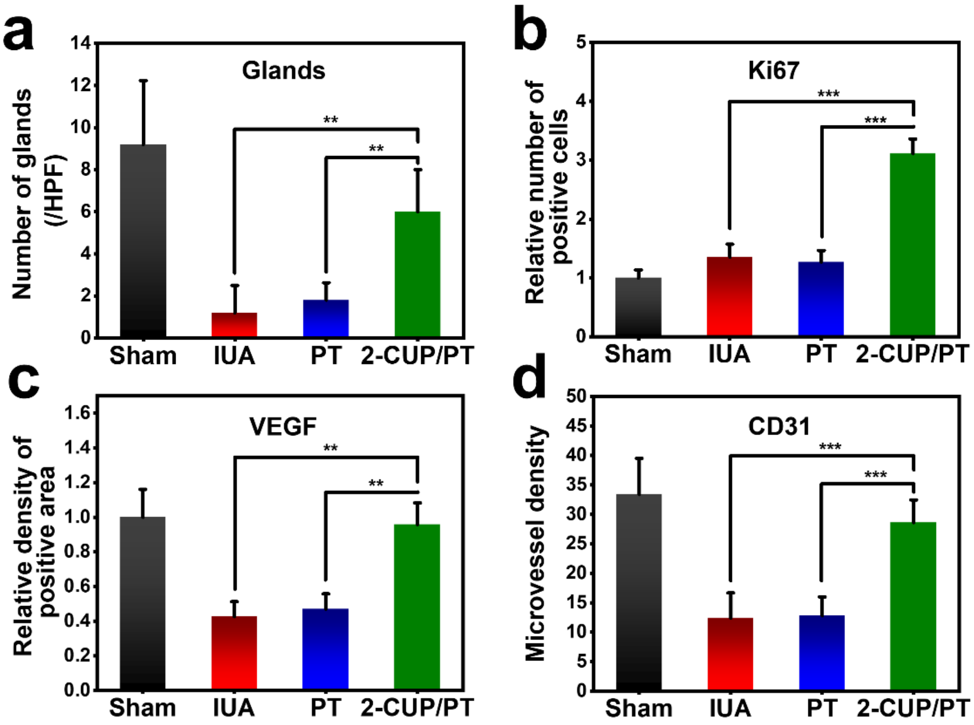

Figure S11. a) The number of glands in endometria after different treatments at day 14 (n=5). b) Relative number of positive cells analyzed from immunohistochemistry staining of Ki67 (n=5). c, d) Quantitative analysis of VEGF (c) and CD31 (d) expression from immunohistochemistry staining images (n=5). All data are presented as mean ± SD. Statistical analysis was performed using one-way ANOVA analysis, $^{**}p < 0.01$, $^{***}p < 0.001$.

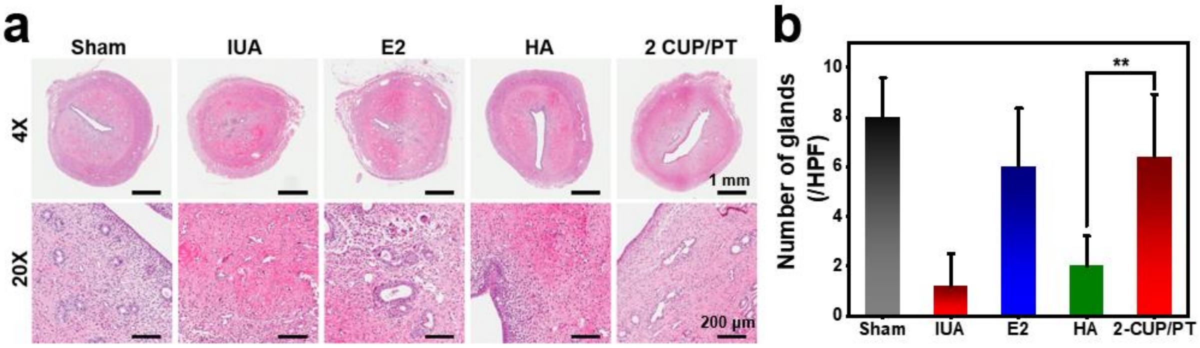

Figure S12. The anti-adhesion and endometrial regeneration ability of CUP/PT composites compared with clinical strategies (hyaluronic acid (HA) gel and 17β-estradiol (E2)). a) Representative H&E staining images of uterine cavity. b) The number of glands in endometria (n=5). All data are presented as mean ± SD. Statistical analysis was performed using one-way ANOVA analysis, $^{**}p < 0.01$.

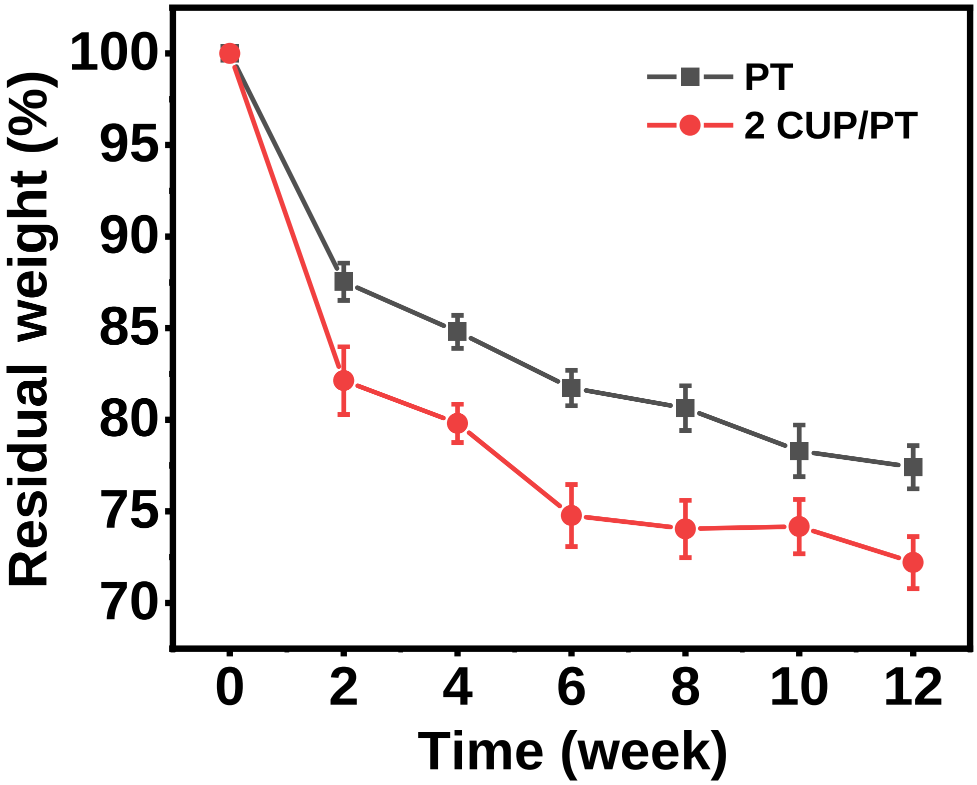

Figure S13. The *in vivo* degradation of PT and 2-CUP/PT composites. All data are presented as mean ± SD, n=3.

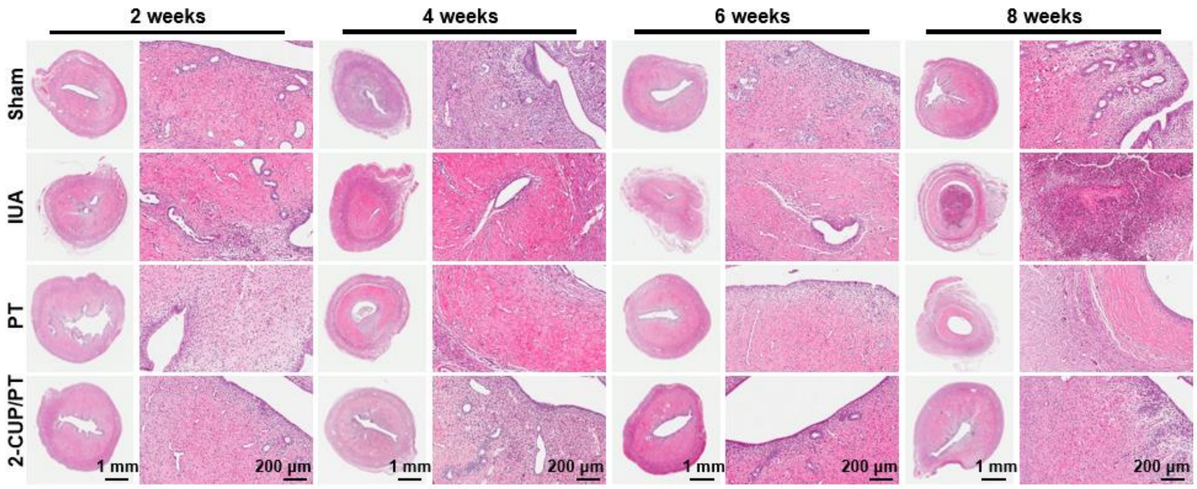

Figure S14. Representative H&E staining images of endometrial regeneration with different treatments at different times (2, 4, 6, and 8 weeks).

.
